# Balloon-Expandable Transcatheter Heart Valve Dislocation Into the Left Ventricle Successfully Repositioned by an “Anchoring Balloon”

**DOI:** 10.1016/j.jaccas.2025.103907

**Published:** 2025-07-09

**Authors:** Mauro Pennacchi, Melwyn Luis Muthukkattil, Marco S. Nazzaro, Davide Cristofani, Francesco De Felice

**Affiliations:** a Department of Cardiology, Interventional Cardiology Unit, San Camillo Hospital, Rome, Italy; b Department of Cardiovascular Sciences, Sapienza University of Rome, Sant’Andrea Hospital, Rome, Italy; c Department of Cardiology, Anesthesiology and Intensive Care Unit, San Camillo Hospital, Rome, Italy

**Keywords:** balloon-expandable valve, complication, transcatheter aortic valve implantation (TAVI), transcatheter repositioning, transfemoral

## Abstract

**Background:**

Transcatheter aortic valve implantation is a well-established treatment option in older patients with symptomatic severe aortic stenosis. Balloon-expandable valve migration is a rare but potentially life-threatening complication.

**Case Summary:**

We report a case of an 87-year-old man with severe aortic stenosis who underwent transfemoral Edwards Sapien 3 valve implantation complicated by delivery-balloon rupture. During deployment, we observed a pressure drop, resulting in incomplete valve expansion. Acute prosthesis migration into the left ventricle occurred. The prosthesis was crossed with a 28-mm VACS-II balloon (OSYPKA) and successfully repositioned by withdrawing the “anchoring balloon.” Angiography showed a well-expanded, well-positioned valve without paravalvular leaks. The patient was discharged 2 days later.

**Discussion:**

To our knowledge, this is the first case ever reported of successful percutaneous recapturing of a balloon transcatheter heart valve dislocated into the left ventricle during transfemoral transcatheter aortic valve implantation.

**Take-Home Message:**

Percutaneous recapturing of dislocated balloon-expandable valve into the left ventricle is feasible and may be a valid bailout strategy.

## Graphical Abstract

**Figure:**
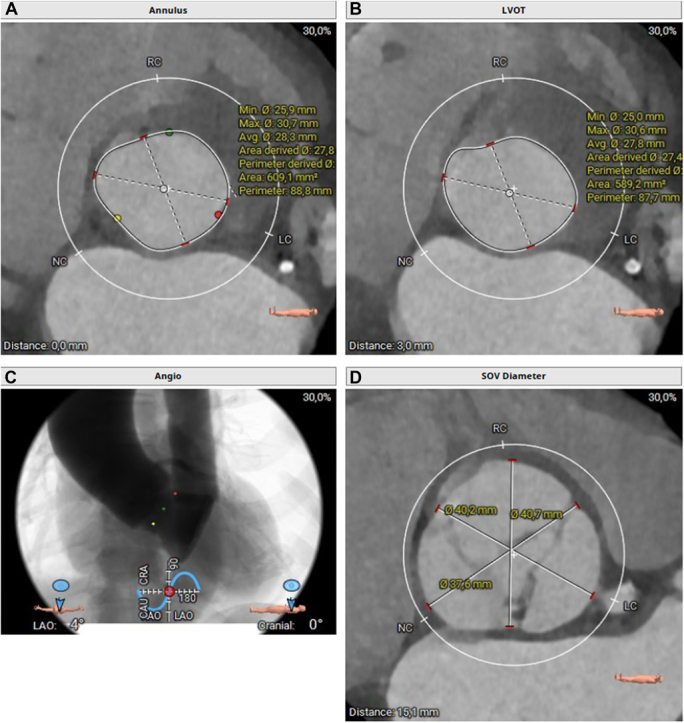

## History of Presentation

An 87-year-old patient was evaluated for mild exertional dyspnea (NYHA functional class III) during a scheduled visit at our outpatient clinic in December 2024.

**Take-Home Message**

• Recapturing and repositioning a balloon-expandable transcatheter heart valve dislocated into the left ventricle is feasible and may represent a therapeutic strategy for this rare complication.

**Learning Objectives**

• To understand the mechanisms of severe balloon THV underexpansion during implantation.
• To understand bailout techniques for recapturing a BEV dislocated into the LV.

## Past Medical History

His personal medical history included arterial hypertension, dyslipidemia, and chronic coronary syndrome with previous percutaneous coronary angioplasty and stenting on the right coronary artery and left circumflex artery.

## Differential Diagnosis

The differential diagnosis in this case is heterogeneous, as it includes chronic coronary syndrome progression; valvular defects such as moderate to severe mitral regurgitation and severe aortic stenosis; endocarditis; and muscular damage as in the case of myocarditis.

## Investigations

A transthoracic echocardiogram showed normal left ventricular dimensions and function with an ejection fraction of 55% as well as a heavily calcified tricuspid aortic valve causing severe aortic stenosis (aortic valve area: 0.8 cm^2^; peak velocity: 4.24 m/s; median pressure gradient: 44 mm Hg; Doppler Velocity Index (DVI): 0.20) and moderate aortic regurgitation (vena contracta: 4 mm; pressure half-time: 440 ms).

**Figure 1:**
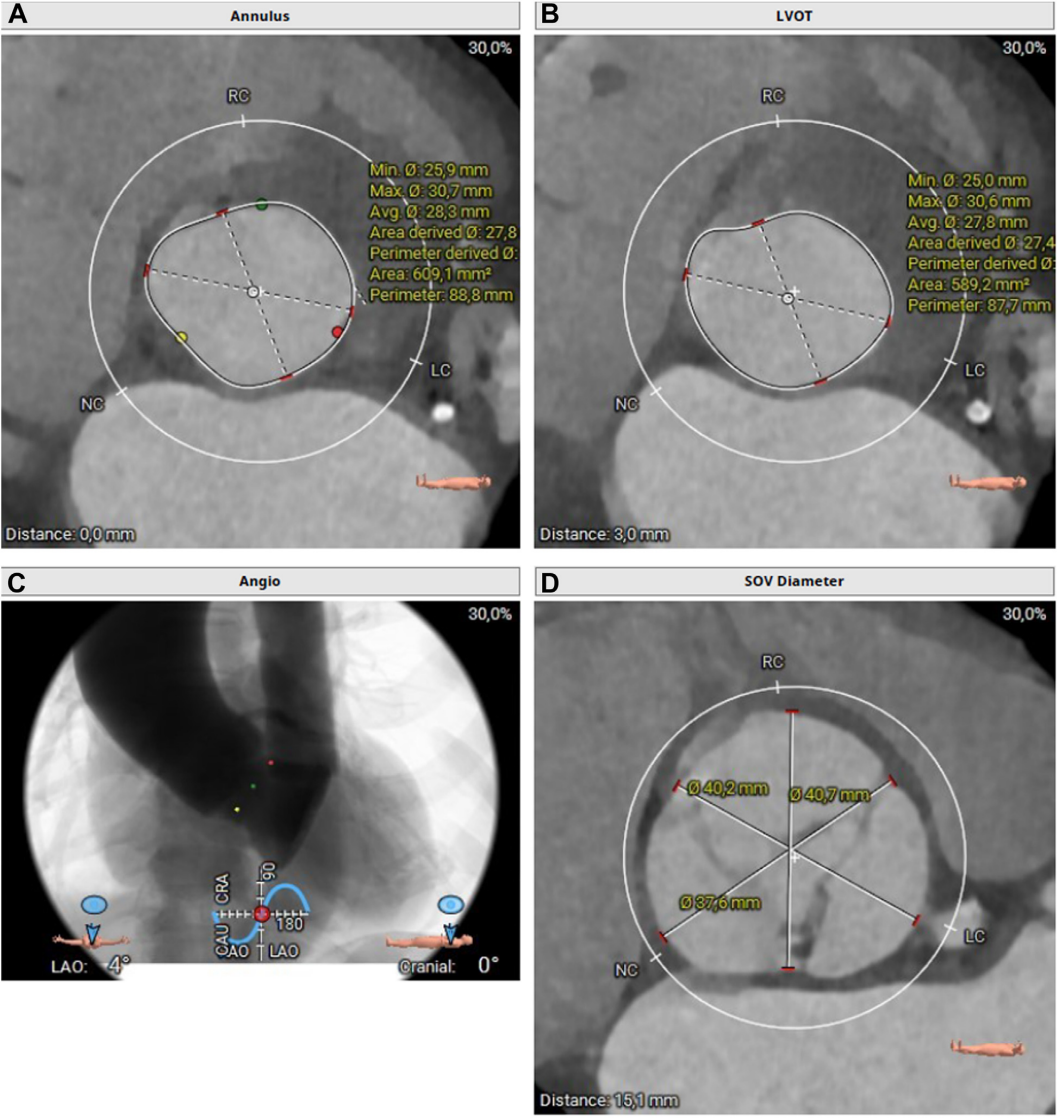
Annular, LVOT, and SOV Measurements on Preoperative CT (A to C) Index CTA images with annular, LVOT, and SOV measurements. (D) CT images of working projections. CT = computed tomography; CTA = computed tomography angiography; LAO = left anterior oblique; LCC = left coronary cusp; LVOT = left ventricular outflow tract; NCC = noncoronary cusp; RCC = right coronary cusp; SOV = sinus of Valsalva.

**Figure 2:**
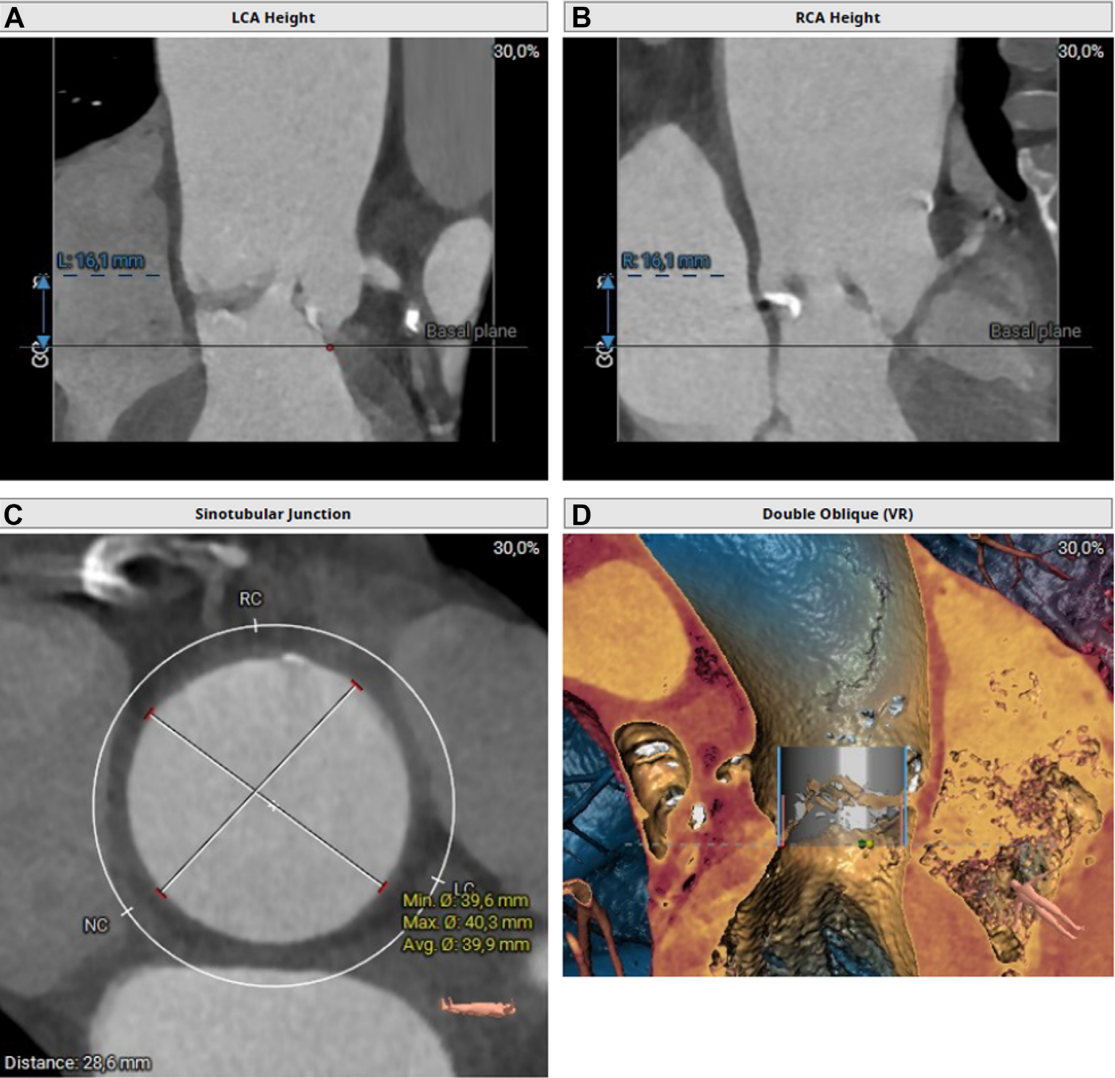
Coronary Heights and Sinotubular Junction Measurements on Preoperative CT (A and B) LCA and RCA heights from the basal plane. (C) Virtual rendering with an Edwards Sapien 3 bioprosthesis in the proper position. (D) Sinotubular junction measurements. CT = computed tomography; LC = left coronary cusp; LCA = left coronary artery; NC = noncoronary cusp; RC = right coronary cusp; RCA = right coronary artery.

**Figure 3:**
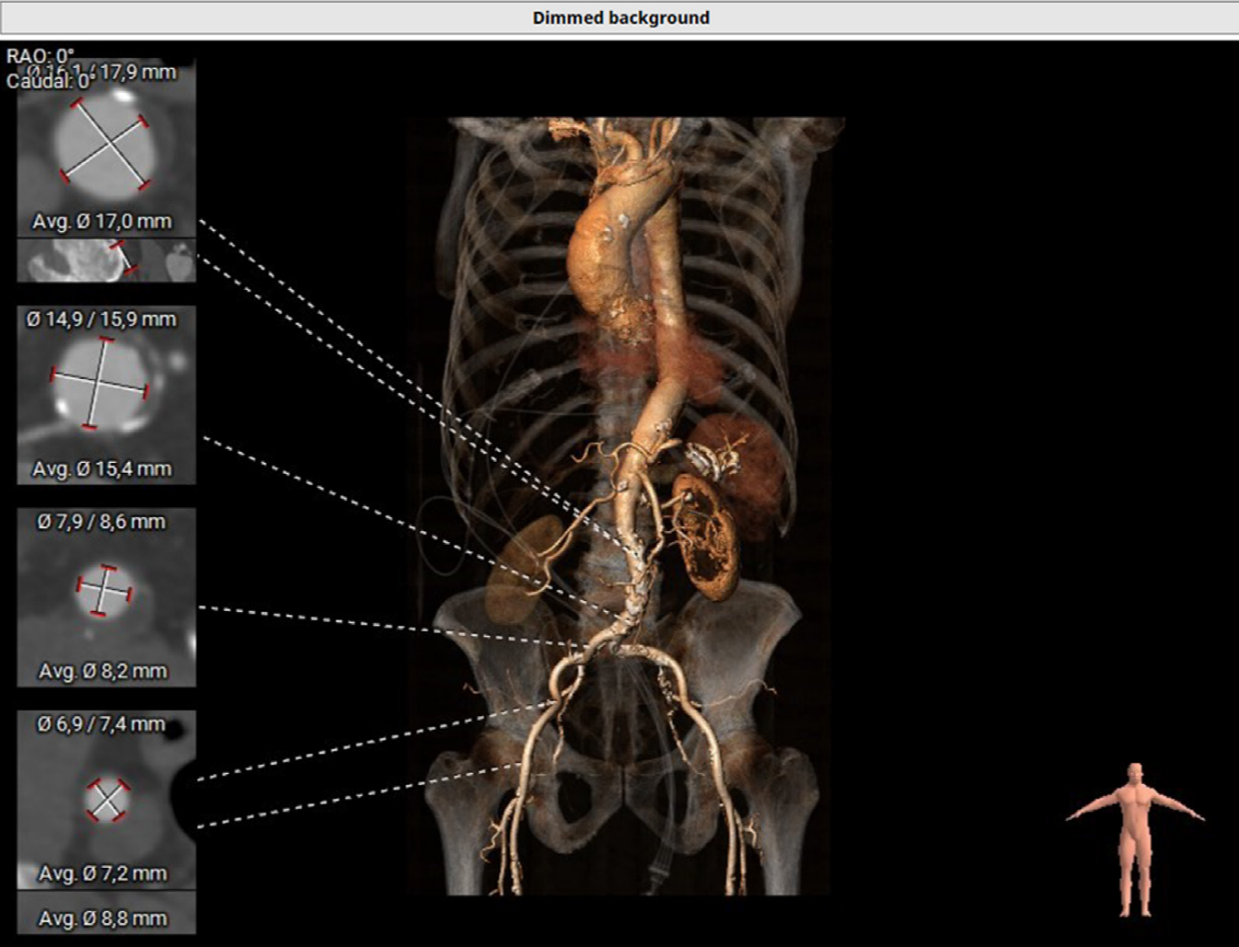
Peripheral Accesses on Preoperative CT Three-dimensional reconstruction of the left and right iliac and femoral arteries, with short-axis measurements. CT = computed tomography; RAO = right anterior oblique.

Preprocedural computed tomography showed mildly calcified aortic valve leaflets with an annulus area of 609 mm^2^ and an annulus diameter of 26 × 30 mm, as well as adequate coronary artery heights and iliac-femoral vascular accesses (Figure 1, Figure 2, Figure 3).

Heart team evaluation favored transcatheter aortic valve implantation (TAVI) of an Edwards Sapien 3 (29 mm) bioprosthesis via transfemoral approach.

## Management

We used a 14-F right femoral artery and a 6-F left radial artery as the primary and secondary accesses, respectively. During rapid left ventricular pacing over the wire, suboptimal delivery balloon expansion was observed (Video 1). Initially, we suspected a possible indeflator-related issue, but a further dilation attempt was unsuccessful. We opted for manual dilation through a syringe containing 50 mL of nondiluted iodine contrast medium, which nonetheless led to suboptimal prosthesis dilation (Video 2). After balloon deflation and withdrawal, acute bioprosthesis migration into the left ventricle occurred, with the stiff wire remaining positioned in the apex (Video 3). We subsequently attempted to recapture the bioprosthesis with a 28-mm NuCLEUS balloon (NuMED) at low atmospheric pressures to prevent complete valvular expansion in the left ventricle. After we anchored the Sapien 3 valve and attempted to reposition it in the distal annulus, additional pulling maneuvers caused slipping of the balloon into the ascending aorta and prosthesis dislocation yet again, without any loss of pacing over the wire. Fortunately, the stiff wire remained fixed inside the prosthesis. While advancing the stiff wire into the left ventricular apex, the prosthesis migrated once again (Video 4). We successfully performed a second retrieval attempt with a 28-mm VACS II balloon during rapid controlled pacing at 140 beats/min, again inflating at low atmospheric pressures, with the aim of temporarily placing the Sapien 3 valve in the annulus. Once correct positioning was confirmed by the pigtail catheter situated in the noncoronary sinus, rapid pacing at 180 beats/min was carried out and the OSYPKA VACS II balloon was fully inflated (Video 5). After repositioning the balloon at the center of the prosthesis, we proceeded with reinflation to optimize valvular expansion (Video 6). Final angiography showed good positioning, with no paravalvular leaks and no pericardial effusion (Video 7). After the procedure, we inspected the delivery balloon and observed contrast leakage from 2 holes at the point where the prosthesis was crimped (Video 8). Hemodynamic parameters were stable and the postprocedural hospital stay was uneventful, with the patient being discharged 2 days later.

## Follow-Up

At 1-month follow-up, the patient reported improvement in dyspnea and the echocardiogram confirmed good Sapien 3 valve positioning without paravalvular leaks and with a mean pressure gradient of 5.4 mm Hg.

## Discussion

Transcatheter aortic valve bioprosthesis dislocation is a rare, possibly life-threatening complication associated with high mortality, with it being the most common cause for emergent cardiac surgery.^1^

To our knowledge, this is the first case reporting successful percutaneous recapturing of a balloon transcatheter heart valve dislocated into the left ventricle during transfemoral TAVI. A previous case report outlined an unsuccessful attempt at retrieving a Sapien XT valve through a valvuloplasty balloon despite extensive pulling maneuvers, which therefore required a snare technique to reposition the valve. Despite repositioning in the annulus, its rotated position determined the moderate to severe paravalvular leak, requiring additional Sapien XT valve implantation distally.^2^ Another case reported Sapien XT valve dislocation into the left ventricular outflow tract (LVOT), requiring urgent deployment of a second Sapien XT valve at a higher position; the operator used the delivery system balloon tip to engage the dislocated valve and carefully pull into back into the LVOT, ensuring it was in a stable coaxial position relative to the second implanted prosthesis.^3^ A further case report has described the use of a valvuloplasty balloon to recapture a balloon-expandable valve dislocated into the left ventricle, but the procedure was performed through a transapical approach and the resulting poor prosthetic position called for second valve implantation.^4^ Existing literature reports regarding self-expanding valve dislocation into the left ventricle have described the use of the “double snare” technique to retrieve and subsequently reposition the valve in the aortic position.^5^

In our case, dislocation into the left ventricle stemmed from delivery system failure and rupture. In our opinion, there could be 2 possible reasons for this failure: either due to a manufacturing defect or due to accidental damage to the balloon during crimping of the prosthesis. The first essential aspect in preventing hemodynamic instability was the constant presence of the stiff wire inside the prosthesis, which allowed sufficient alignment with the LVOT and prevented malrotation, a condition that can be fatal. The second aspect is the choice of the balloon for anchoring and retrieving the valve. Despite initially using the NuMED NuCLEUS balloon, which appeared more suitable due to its dog-bone shape, the OSYPKA VACS II balloon was able to both anchor and correctly retrieve the valve because of the lower profile of the balloon. Furthermore, valve underexpansion allowed less encumbrance during the retrieval phase and positioning. Stable pigtail catheter positioning in the noncoronary sinus also proved key, as it provided a useful marker for correct implantation. Lastly, in our opinion, controlled pacing over the wire appears to have been paramount in recapturing the valve, modulating it at 120/140 beats/min during the retrieving phase to reduce ventricular contraction boost.

## Conclusions

To our knowledge, this case is the first report of successful recapturing and repositioning of a balloon-expandable transcatheter aortic valve dislocated into the left ventricle during transfemoral TAVI.

## Funding Support and Author Disclosures

The authors have reported that they have no relationships relevant to the contents of this paper to disclose.

## Appendix

Video 1 Suboptimal Delivery Balloon Expansion During Rapid Left Ventricular Pacing

Video 2 Suboptimal Prosthesis Expansion During Manual Dilation

Video 3 Acute Bioprosthesis Migration Into the Left Ventricle After Balloon Withdrawal

Video 4 NuCLEUS Balloon Slippage and Prosthesis Migration During Stiff Wire Advancement Into the Left Ventricular Apex

Video 5 Correct Prosthesis Positioning and VACS-II Balloon Inflation During Rapid Pacing

Video 6 Optimizing Valvular Expansion Through Balloon Reinflation

Video 7 Final Angiography Showing Good Prosthesis Positioning, With No Paravalvular Leaks and No Pericardial Effusion

Video 8 Contrast Leakage From the Delivery Balloon

## References

[1] Eggebrecht H., Schmermund A., Kahlert P., Erbel R., Voigtländer T., Mehta R.H. (2013). Emergent cardiac surgery during transcatheter aortic valve implantation (TAVI): a weighted meta-analysis of 9,251 patients from 46 studies. EuroIntervention.

[2] Dumonteil N., Marcheix B., Grunenwald E., Roncalli J., Massabuau P., Carrié D. (2013). Left ventricular embolization of an aortic balloon-expandable bioprosthesis: balloon capture and reimpaction as an alternative to emergent conversion to open-heart surgery. JACC Cardiovasc Interv.

[3] Nicolino A., Vischi M., Moshiri S. (2014). Valve migration into the left ventricular outflow tract managed by coaxial double-valve alignment. JACC Cardiovasc Interv.

[4] Tiroch K., Schleiting H., Karpettas N. (2015). How should I treat dislocation of a TAVI SAPIEN prosthesis into the left ventricle?. EuroIntervention.

[5] Bunc M., Vitez L., Terseglav S., Žlahtič T., Ussia G.P. (2021). Transcatheter aortic valve dislocation in left ventricular outflow tract with successful repositioning using “double snare” technique. Int J Clin Med.

